# Supplementary material for: Epidemiological Studies of Pan-Azole Resistant Aspergillus fumigatus Populations Sampled during Tulip Cultivation Show Clonal Expansion with Acquisition of Multi-Fungicide Resistance as Potential Driver
Source: Microorganisms. 2021 Nov 18;9(11):2379. doi: 10.3390/microorganisms9112379 (PMC8618125; doi:10.3390/microorganisms9112379)
Supplement: Supplementary file 1 [file microorganisms-09-02379-s001.zip › microorganisms-1463567-supplementary.pdf]

Table S1. Isolates characterised using STR*Af* typing and sequence analysis of genes encoding CSP and CYP51A. Product sizes (bp) are presented for STR*Af* markers 2A, 2B, 2C, 3A, 3B, 3C, 4A, 4B and 4C.

Isolate[1]	**Origin**	**Location**	**Year**	**CYP51A variant**	**2A**	**2B**	**2C**	**3A**	**3B**	**3C**	**4A**	**4B**	**4C**	**CSP type**
Air teb1	Air	Netherlands	2018	TR_{34}/L98H/T289A/G448S	188	123	158	136	163	82	180	185	226	t02?
HPPb-1*	Air	UK	2018	TR_{34}/L98H	192	143	175	202	160	94	188	201	166	t02
BTTa-1*	Air	Netherlands	2018	TR_{46}/Y121F/M172I/T289A/G448S	194	146	164	187	163	82	200	181	226	t01
Air carb5	Air	Netherlands	2018	wt	194	143	164	202	160	85	180	185	266	t02
BKCb-1*	Air	Netherlands	2018	TR_{46}/Y121F/T289A/S363P/I364V/G448S	194	143	164	238	166	97	196	181	182	t01
V094-26	Patient	Netherlands	2010	TR_{46}/Y121F/T289A	163	143	164	214	160	94	196	181	182	t01
CXH_07	Patient	UK		TR_{34}/L98H	171	149	171	187	169	91	180	209	246	t04A
ASP251	Patient	Germany		TR_{34}/L98H/S297T/F495I	171	123	158	199	160	82	180	185	226	t02
OKH50	Patient	Japan		TR_{34}/L98H	171	146	156	202	160	82	180	185	226	t02
ARAF017	Patient	Netherlands		TR_{34}/L98H	171	143	173	202	166	94	180	201	226	t04A
ARAF013	Patient	Netherlands		TR_{34}/L98H/S297T/F495I	171	143	156	229	160	97	180	185	226	t11
D007	Patient	Taiwan		TR_{34}/L98H/S297T/F495I	181	135	164	196	240	94	180	185	166	t04A
CYP_15_38	Patient	Belgium		TR_{46}/Y121F/T289A	182	146	173	232	160	94	180	185	182	t09
ASP267	Patient	Germany		TR_{34}/L98H	182	123	164	247	163	91	180	173	190	t11
CXH_06	Patient	UK		TR_{34}/L98H	182	123	164	284	163	97	180	181	190	t04B
CYP_15_63	Patient	Belgium		TR_{34}/L98H/S297T/F495I	188	123	158	223	172	94	196	185	186	t01
ASP168	Patient	Germany		TR_{34}/L98H	188	146	171	376	169	85	215	181	186	t04B
CYP_15_46	Patient	Belgium		TR_{34}/L98H	192	143	156	208	160	94	180	185	166	t02
ASP164	Patient	Germany		TR_{34}/L98H	192	125	164	382	160	88	180	185	178	t11
CYP_15_2	Patient	Belgium		TR_{46}/Y121F/T289A	194	143	164	205	160	97	196	181	226	t01
CYP_15_7	Patient	Belgium		TR_{46}/Y121F/T289A	194	146	158	208	166	130	180	201	186	t01
V093-54	Patient	Netherlands	2009	TR_{46}/Y121F/T289A	194	146	158	208	166	130	180	201	186	t01
CYP_15_80	Patient	Belgium		TR_{46}/Y121F/T289A	194	143	156	235	160	82	180	185	226	t02
AF65*	Reference	UK	1997	wt	171	143	164	214	160	94	180	185	230	t02
WT-NL*	Reference	Netherlands	<2014	wt	188	141	169	262	166	85	200	181	166	t01
08-19-02-10*	Reference	Netherlands	2008	TR_{34}/L98H	192	152	164	364	160	91	180	185	190	t04B

TR34-NL*	Reference	Netherlands	<2014	TR_{34}/L98H	192	152	164	364	160	91	180	185	190	
TR46-NL*	Reference	Netherlands	<2014	TR_{46}/Y121F/T289A	194	146	164	187	160	124	203	181	182	
AF293*	Reference	UK	1993	F46Y/M172V/N248T/D255E/E427K	194	139	175	247	195	133	192	185	178	t06A
F2-B4	Soil	France	2016	F46Y/M172V/E427K	163	131	160	160	166	109	176	165	166	t07
UK5-A9	Soil	UK	2016	F46Y/M172V/E427K	163	137	160	178	172	127	176	165	170	t08
BB1-2.2-A4	Soil	UK	2015		169	146	162	202	211	88	188	181	178	t18A
PG3-8	Soil	UK	2015	wt	171	143	156	214	160	94	180	185	226	t02
SS8-7A*	Soil	Belgium	2015	TR_{34}/L98H	182	146	171	343	169	97	215	189	230	t11
G5-C5	Soil	Germany	2016	wt	188	141	171	436	169	115	215	181	186	t13
RN8-18*	Soil	UK	2016	TR_{34}/L98H	192	152	164	364	160	91	180	185	190	t04B
PG3-4	Soil	UK	2015		196	139	169	130	166	156	253	185	178	
F5-C6*	Soil	France	2016	F46Y/M172V/E427K	163	132	160	160	172	106	176	165	166	t15
BB1-3-B9*	Soil	UK	2015	F46Y/M172V/E427K	163	133	160	160	172	88	176	165	170	t02*
G1-A9*	Soil	Germany	2016	F46Y/M172V/E427K	163	134	160	160	172	88	176	165	170	t02*
SS5-7C*	Soil	Austria	2015	F46Y/M172V/E427K	163	135	160	160	172	124	176	165	170	t08
BB6-8-A9	Soil	UK	2015	F46Y/M172V/E427K	163	135	162	160	172	106	176	165	170	t08
BB6-8-C7*	Soil	UK	2015	F46Y/M172V/E427K	163	133	160	166	169	100	176	165	170	t19
UK2-B4*	Soil	UK	2016	F46Y/M172V/E427K	163	133	160	187	166	88	176	165	166	t08
RS3-3*	Soil	UK	2016	TR46/Y121F/T289A	163	143	156	235	160	94	196	185	226	t02
WN19-3*	Soil	UK	2016	TR46/Y121F/T289A	163	141	164	244	160	216	180	185	186	t01
SS10-10A	Soil	Netherlands	2015	wt	169	148	158	136	163	85	180	181	222	t02
G2-C1	Soil	Germany	2016	wt	169	143	160	136	163	94	180	181	226	t05
BB1-3-C8	Soil	UK	2015	wt	169	123	158	178	166	85	180	181	234	t11
BB1-2.2-B1*	Soil	UK	2015	wt	169	147	162	202	211	88	188	181	178	t18A
BB1-2.2-A10	Soil	UK	2015	wt	169	148	162	202	211	88	188	181	178	t18A
UK5-B5*	Soil	UK	2016	TR_{34}/L98H	171	143	156	202	160	94	180	185	190	t02
UK5-C4	Soil	UK	2016	wt	171	143	162	217	160	85	180	185	194	t02
PG2-6	Soil	UK	2015	D262Y	173	143	158	136	163	82	180	185	186	t05
SS7-2A	Soil	Germany	2015	L27I	173	141	166	150	163	124	180	185	166	t03
SS5-2A*	Soil	Austria	2015	D262Y	179	127	158	136	163	100	180	185	174	t05

PG1-5	Soil	UK	2015	wt	179	127	171	187	163	127	180	177	166	t03
PG2-10*	Soil	UK	2015	wt	179	127	156	190	163	118	184	181	166	t04B
BB6-8-C10	Soil	UK	2015	wt	179	127	156	193	163	127	184	181	166	
PG3-9	Soil	UK	2015	wt	179	127	156	196	163	136	184	181	178	t04A
SS10-6A*	Soil	Netherlands	2015	TR_{34}/L98H	182	146	171	157	169	85	216	241	282	t11
BB6-8-B1*	Soil	UK	2015	D262Y	182	141	156	217	175	130	184	181	166	t03
WN28-6*	Soil	UK	2016	TR_{46}/Y121F/T289A	182	143	168	256	160	175	180	181	182	t01
F3-C6	Soil	France	2016	wt	184	148	175	187	163	109	184	197	178	t03
SSB8-9A	Soil	Belgium	2015	wt	184	123	162	217	200	94	216	241	166	t05
G2-B8	Soil	Germany	2016	wt	188	150	179	217	192	140	207	185	178	t10
BB1-8-A2	Soil	UK	2015	wt	188	143	169	220	166	85	219	249	166	t04A
BB1-8-A5	Soil	UK	2015	wt	192	143	158	136	163	94	180	185	186	t05
PG3-6	Soil	UK	2015	wt	192	143	156	208	160	88	180	185	230	t06A
F1-B1	Soil	France	2016	wt	192	141	171	134	169	184	176	181	186	t13
BB1-8-A7	Soil	UK	2015	wt	194	148	177	190	160	136	188	181	166	t01
PG3-3*	Soil	UK	2015	wt	196	139	169	130	166	159	253	185	178	t01
T8-2	Tulip bulbs	UK	2015	wt	169	127	185	160	172	139	188	185	166	t11
T2-4	Tulip bulbs	Netherlands	2015	wt	179	141	162	153	163	175	180	181	166	t03
T4-1	Tulip bulbs	Netherlands	2015	F46Y/M172V/E427K	163	137	160	160	166	104	176	165	170	t08
T6-1	Tulip bulbs	UK	2015	F46Y/M172V/E427K	163	133	160	184	166	127	176	165	166	t13
T1-3	Tulip bulbs	Netherlands	2015	wt	169	143	160	133	163	94	180	181	226	t05
T11-8	Tulip bulbs	Netherlands	2017	TR_{34}/L98H	171	143	158	202	160	94	180	185	258	t02
T11-B2	Tulip bulbs	Netherlands	2017	TR_{34}/L98H	171	143	158	202	160	94	180	185	262	t02
T6-5	Tulip bulbs	UK	2015	wt	171	143	164	238	160	85	180	193	308	t02
T5-8	Tulip bulbs	Netherlands	2015		177	127	162	150	163	130	180	177	166	
T3-3	Tulip bulbs	Netherlands	2015	TR_{34}/L98H	182	146	171	343	169	97	215	189	230	t11
T3-6	Tulip bulbs	Netherlands	2015	TR_{34}/L98H	182	146	171	343	169	97	215	189	230	t11
T5-3	Tulip bulbs	Netherlands	2015	TR_{34}/L98H	182	146	171	343	169	97	215	189	230	t11
T10-5	Tulip bulbs	UK	2015	TR_{34}/L98H	182	146	171	343	169	97	215	189	230	t11
T2-8	Tulip bulbs	Netherlands	2015	TR_{34}/L98H	182	146	171	343	169	97	215	189	230	t11

T1-2	Tulip bulbs	Netherlands	2015	TR_{34}/L98H	182	146	171	343	169	97	215	189	230	t11
T6-3	Tulip bulbs	UK	2015	TR_{34}/L98H	182	146	171	343	169	97	215	189	230	t11
T4-7	Tulip bulbs	Netherlands	2015	TR_{34}/L98H	182	146	171	343	169	97	215	189	230	t11
T2-1	Tulip bulbs	Netherlands	2015	TR_{34}/L98H	182	148	171	343	169	97	215	189	230	t11
T10-6	Tulip bulbs	UK	2015		188	143	169	220	166	85	219	249	166	
T9-2	Tulip bulbs	UK	2015	wt	188	141	177	550	160	118	168	189	186	t01
T1-4	Tulip bulbs	Netherlands	2015	wt	190	123	156	172	160	85	208	181	202	t06B
T5-6	Tulip bulbs	Netherlands	2015	wt	192	143	164	208	160	85	180	185	234	t02
T4-10	Tulip bulbs	Netherlands	2015	TR_{46}/Y121F/T289A	194	146	164	187	160	124	203	181	182	t01
T5-1	Tulip bulbs	Netherlands	2015	TR_{46}/Y121F/T289A	194	146	164	187	160	124	203	181	182	t01
T5-2	Tulip bulbs	Netherlands	2015	TR_{46}/Y121F/T289A	194	146	164	187	160	124	203	181	182	t01
T3-5	Tulip bulbs	Netherlands	2015	TR_{46}/Y121F/T289A	194	146	164	187	160	124	203	181	182	t01
T7-9	Tulip bulbs	UK	2015	TR_{46}/Y121F/T289A	194	146	164	187	160	124	203	181	182	t01
T5-5	Tulip bulbs	Netherlands	2015	TR_{46}/Y121F/T289A	194	146	164	187	160	124	203	181	182	t01
T4-9	Tulip bulbs	Netherlands	2015	TR_{46}/Y121F/T289A	194	146	164	187	160	124	203	181	182	t01
TP UT4A-1	Tulip peel waste	Netherlands	2018	TR_{46}/Y121F/T289A	194	146	164	187	160	124	203	181	182	t01
TP UT4B-2	Tulip peel waste	Netherlands	2018	TR_{46}/Y121F/T289A	194	146	164	187	160	124	203	181	182	t01
TP UT5C-5	Tulip peel waste	Netherlands	2018	TR_{34}/L98H	171	146	156	202	160	82	180	185	226	t02
TP UT1C-1	Tulip peel waste	Netherlands	2018	wt	182	152	162	226	172	159	224	181	166	t03
TP UT1B-1	Tulip peel waste	Netherlands	2018	TR_{34}/L98H	182	143	164	324	160	94	180	185	166	t11
TP UT4B-1	Tulip peel waste	Netherlands	2018	TR_{34}/L98H	182	143	164	324	160	94	180	185	166	t11
TP TEB5C-2	Tulip peel waste	Netherlands	2018	TR_{34}/L98H/T289A/I364V/G448S	188	123	164	136	166	82	196	185	226	t02
TP UT5C-7	Tulip peel waste	Netherlands	2018	TR_{34}/L98H	188	123	158	223	163	79	180	193	226	t02
TP UT1A-7	Tulip peel waste	Netherlands	2018	wt	188	141	169	262	166	85	200	181	166	t01
TP UT1A-2	Tulip peel waste	Netherlands	2018	TR_{34}/L98H	192	146	171	157	169	91	180	241	190	t02
TP TEB5C-3	Tulip peel waste	Netherlands	2018	TR_{46}/Y121F/T289A	192	143	158	244	160	97	180	185	182	t02
TP UT5B-9	Tulip peel waste	Netherlands	2018	TR_{46}/Y121F/T289A??	194	123	164	202	160	82	180	201	182	t02
TP UT5B-3	Tulip peel waste	Netherlands	2018	TR_{46}/Y121F/T289A	163	143	156	238	160	94	196	185	226	t02
STNL4-B6	Tulip soil	Netherlands	2016		163	133	160	184	166	127	176	165	166	
STNL6-B2	Tulip soil	Netherlands	2016	TR_{34}/L98H	182	123	156	253	163	94	180	173	190	

STNL5-B6	Tulip soil	Netherlands	2016	TR$_{34}$/L98H	188	146	164	355	160	85	188	181	230	t11
STNL5-C8	Tulip soil	Netherlands	2016	TR$_{46}$/Y121F/T289A	194	146	158	208	166	130	180	201	186	
STNL2-C9	Tulip soil	Netherlands	2016	F46Y/M172V/E427K	163	135	160	157	172	130	176	165	166	t02*
STNL5-C5	Tulip soil	Netherlands	2016	TR$_{34}$/L98H/S297T/F495I	171	123	158	202	160	82	180	201	226	t02
STNL6-B1	Tulip soil	Netherlands	2016	TR$_{34}$/L98H	171	146	156	205	160	94	180	185	226	t01
STNL1-A8	Tulip soil	Netherlands	2016	wt	177	139	181	208	178	85	176	209	178	t04A
STNL2-B8	Tulip soil	Netherlands	2016	TR$_{34}$/L98H	182	146	164	202	160	97	180	181	222	t04B
STNL6-A3	Tulip soil	Netherlands	2016	TR$_{34}$/L98H	188	148	166	302	160	88	180	181	190	t01
STNL5-B7	Tulip soil	Netherlands	2016	TR$_{46}$/Y121F/T289A	192	143	164	244	160	82	196	185	182	t02
STNL3-C8	Tulip soil	Netherlands	2016	TR$_{34}$/L98H	192	143	164	343	163	94	180	181	166	t04B
STNL5-C1	Tulip soil	Netherlands	2016	TR$_{46}$/Y121F/T289A	194	135	160	208	160	104	180	201	186	t06A

[1] Isolates marked with asterisk have been characterised in previous study [17]
